# Understanding ChatGPT adoption among medical students: an integrated theoretical model of psychological barriers and individual factors

**DOI:** 10.3389/fmed.2026.1945510

**Published:** 2026-10-09

**Authors:** Ayoob Lone, Ali Saleh Alshebami, Imtiyaz Ahmad Dar, Naved Iqbal

**Affiliations:** 1 Department of Clinical Neurosciences, College of Medicine, King Faisal University, Al-Ahsa, Saudi Arabia; 2 Applied College, King Faisal University, Al-Ahsa, Saudi Arabia; 3 Department of Psychology, University of Kashmir, Hazratbal Srinagar, J&K, India; 4 Department of Psychology, Jamia Millia Islamia, New Delhi, India

**Keywords:** artificial intelligence, behavioral intention, ChatGPT adoption, guilt feelings, innovativeness, medical students, PLS-SEM, privacy concern

## Abstract

**Background:**

The growing adoption of generative artificial intelligence (GenAI), particularly ChatGPT, has created new opportunities for medical education by supporting learning, problem-solving, and knowledge acquisition. However, evidence regarding the psychological and individual factors influencing ChatGPT adoption among medical students remains limited. This study develops and tests an integrated theoretical model that combines Privacy Theory, Technostress Theory, Affect Theory, Diffusion of Innovation Theory, and the Theory of Planned Behavior to explain ChatGPT adoption.

**Material and methods:**

A quantitative cross-sectional study was conducted among undergraduate medical students at King Faisal University, Saudi Arabia using a structured, self-administered questionnaire. The proposed model examined the effects of privacy concern, technophobia, guilt feelings, and innovativeness on behavioral intention and ChatGPT usage behavior, with behavioral intention serving as the mediating variable. Data were analyzed using Partial Least Squares Structural Equation Modeling (PLS-SEM).

**Results:**

The findings showed that innovativeness had a significant positive relationship with behavioral intention (*β* = 0.579, *p* < 0.001), while behavioral intention had a strong positive relationship with ChatGPT usage behavior (*β* = 0.777, *p* < 0.001). In addition, behavioral intention significantly mediated the relationship between innovativeness and ChatGPT usage behavior (*β* = 0.450, *p* < 0.001). In contrast, privacy concern, technophobia, and guilt feelings were not significantly related to behavioral intention, and their indirect relationships with ChatGPT usage behavior were also not significant.

**Conclusion:**

Within the proposed model, personal innovativeness was the only antecedent significantly associated with behavioral intention, whereas the psychological barriers were not statistically significant. By integrating five complementary theoretical perspectives into a single framework, this study extends current understanding of generative AI adoption in medical education and highlights behavioral intention as the key mechanism linking individual characteristics to actual use. The findings provide theoretical insights and practical guidance for promoting the responsible and effective integration of ChatGPT into medical education through initiatives that foster innovativeness and AI literacy.

## Introduction

Artificial intelligence (AI) has become an integral part of contemporary society, transforming diverse sectors including healthcare (1), business (2), and education (3). In addition, Generative Artificial Intelligence (GenAI), which allows systems to create human-like text, communicate with people in a natural way and help in completing higher order cognitive tasks (4, 5), represents the newest step forward in AI development. Unlike earlier technologies, which helped in information searching, Gen AI actually helps in information generation and solving problems; therefore, students are able to interact with educational material in a more flexible and individual way (6, 7). As a result, it became one of the most influential recent technological achievements in the sphere of higher education, attracting increased attention from educators, researchers, and policy makers. In this context, one of the most popular among the fast-developing GenAI applications is ChatGPT developed by OpenAI. By means of natural language interactions, ChatGPT can help to understand complex concepts, summarize learning materials, help with academic writing, create learning resources, and provide feedback (8–11). In this way, it contributes to more active engagement with course material and facilitates independent learning.

The fast-growing popularity of ChatGPT has raised much interest concerning its educational potential. Some research indicates that generative AI can increase the efficiency of the learning process, support self-directed learning, and promote critical thinking when used properly (10, 11). Simultaneously, however, the recent systematic review of the literature indicates that the educational potential of ChatGPT not only depends on its technical features but also on willingness of students to use and integrate it into their learning (6, 7). For this reason, the identification of the factors that contribute to ChatGPT adoption is an urgent research task.

The relevance of ChatGPT is especially apparent in the field of medical education, where students are expected to master an ever-expanding body of scientific knowledge while developing clinical reasoning and evidence-based decision-making skills (12–14). In addition, medical training is associated with high workload, constant assessments, and increased exposure to digital technologies, which create opportunities for complementary learning using AI technology. Recent studies have shown, large language models show good performance in a number of medical knowledge assessments, which proves their ability to contribute to but not replace traditional medical education (15).

However, it is evident that different students demonstrate different attitudes towards using ChatGPT. Some students easily adopt generative AI as a learning tool whereas others are not ready to use it despite being aware of its educational value. Thus, besides the technical features of the technology, it is necessary to consider the psychological reactions of students to generative AI as well as their individual characteristics (16, 17). One of the factors that attracted much attention recently is the issue of privacy concern, technophobia, guilt feelings, and personal innovativeness. Privacy concern can make students refrain from interacting with AI because they do not want to share academic information with a machine (18, 19). Technophobia can reduce willingness to use new technologies because of lack of confidence in them (20, 21). Guilt feelings can occur because students feel that using AI in learning contradicts academic integrity and their personal beliefs (22). At the same time, those who demonstrate high level of personal innovativeness are more willing to experiment with new technologies (23).

Taken together, these factors suggest that students’ willingness to adopt ChatGPT may reflect the interplay between potential psychological barriers and individual openness to technological innovation. One set of factors may discourage adoption because of perceived risk, uncertainty, or ethical concerns associated with privacy concern, technophobia, and guilt feelings. In contrast, personal innovativeness may facilitate willingness to experiment with and adopt emerging technologies.

Despite the growing body of research on ChatGPT and other generative AI technologies in higher education, important gaps remain in understanding the factors that shape students’ adoption of these technologies. Existing literature has primarily used traditional technology acceptance theories, such as Technology Acceptance Model (TAM), Unified Theory of Acceptance and Use of Technology (UTAUT), and the Theory of Planned Behavior (TPB), focusing mainly on cognitive predictors like perceived usefulness, perceived ease of use, and performance expectancy (24–28). Even though such models have contributed significantly to the understanding of ChatGPT adoption, the explanation of students’ adoption behaviors is partial, as the psychological and emotional factors influencing the process of using technology are considered only to a small extent. In particular, such constructs as privacy concern, technophobia, and guilt feelings are insufficiently researched even though the issues of privacy, anxiety related to technology, and ethical problems related to AI assistance in learning have become topical (18–22, 29–31). Similarly, even though the construct of personal innovativeness has been acknowledged as a significant predictor of technology adoption, its role in students’ acceptance of generative AI, especially in connection with psychological barriers, has been relatively under-researched (23, 32–35).

A second limitation concerns the research context. Although most studies investigating ChatGPT adoption were based on samples of general population of university students or multidisciplinary students, there are only limited amounts of data available for medical education (12–15). This represents an important gap as the medical education is characterized by specific requirements including continuous learning, decision making based on evidence, professional ethics, and responsible handling of confidential information. Such characteristics may influence students’ perception of both the possibilities and risks of using generative AI technologies. Moreover, empirical evidence for Saudi Arabia, where significant investments in AI and digital transformation have been made through Saudi Vision 2030 as national strategy (36) is relatively lacking.

In order to fill these gaps, the present study proposes an integrated theoretical model based on The Privacy Paradox framework (29), Technostress Theory (30), Affect Theory (31), Diffusion of Innovation Theory (32), and the Theory of Planned Behavior (24). The rationale for this integration is that ChatGPT adoption among medical students is unlikely to be explained adequately by a single theoretical tradition because adoption simultaneously involves perceived risks, technology-related psychological responses, affective and moral evaluations, and individual differences in openness to innovation. Each theoretical perspective contributes a theoretically distinct explanation. A privacy-risk perspective explains how concerns about the potential consequences of disclosing information to an AI system may discourage adoption. Technostress Theory provides a broader explanation of how technology-related complexity, uncertainty, and perceived lack of control can generate psychological discomfort, while research specifically addressing technophobia provides the direct conceptual basis for the present construct. A cognitive-appraisal perspective explains how students’ evaluations of ChatGPT use in relation to their personal and academic standards may generate guilt and consequently influence willingness to use the technology. Diffusion of Innovation Theory explains why students differ in their predisposition to experiment with and adopt emerging technologies. Finally, TPB provides the conceptual basis for the intention–behavior linkage by positioning behavioral intention as a proximal motivational construct associated with subsequent technology-use behavior. The present study draws specifically on this intention–behavior component of TPB rather than applying TPB as a complete theoretical framework, because the canonical TPB predictors of attitude, subjective norm, and perceived behavioral control were not included in the present model.

The proposed framework was intentionally specified as an intention–behavior pathway rather than as a comprehensive model containing all possible direct effects on ChatGPT usage. Privacy Concern, Technophobia, Guilt Feeling, and Innovativeness were conceptualized as antecedent factors that may shape students’ Behavioral Intention to use ChatGPT, whereas Behavioral Intention was positioned as the proximal intention construct linking these antecedents to self-reported ChatGPT Usage Behavior. Accordingly, the present model estimates the relationships between the antecedents and Behavioral Intention, the relationship between Behavioral Intention and ChatGPT Usage Behavior, and the corresponding specific indirect effects through Behavioral Intention. This specification does not imply that the antecedent variables cannot have direct associations with usage; rather, direct antecedent-to-usage paths were outside the scope of the theoretically specified model and were not estimated in the present study. Therefore, any indirect effects identified in this model are interpreted as specific indirect associations through Behavioral Intention rather than as evidence of a particular conventional mediation type.

Accordingly, the theoretical integration occurs at the level of the adoption process rather than through theoretical equivalence. The four antecedent domains represent different psychological and individual routes through which students may form an intention to use ChatGPT, whereas behavioral intention provides a common motivational endpoint that links these antecedents to reported usage behavior. This architecture allows the study to examine whether facilitating and inhibiting influences originating from different theoretical traditions converge in students’ willingness to use ChatGPT and whether this willingness is subsequently reflected in their reported use. The framework should therefore be understood as a theoretically informed integration of complementary perspectives rather than as a claim to have developed a new unified theory of technology adoption. The conceptual framework guiding the study is presented in Figure 1. The theoretical rationale underlying each of these relationships is developed in the following section.

**Figure 1:**
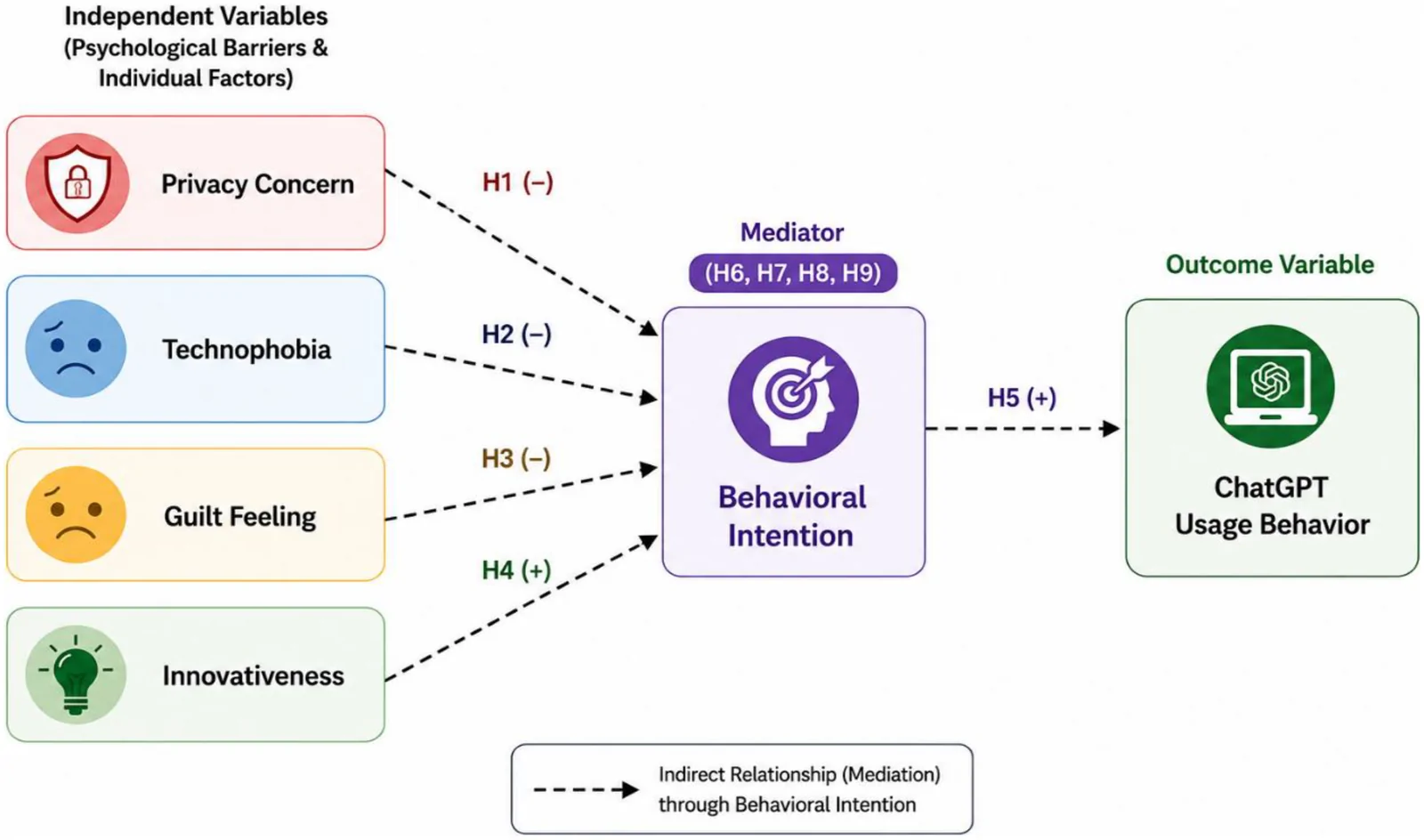
Integrated theoretical framework of ChatGPT adoption among undergraduate medical students.

### Theoretical framework and hypothesis development

Building on the conceptual framework presented in Figure 1, the proposed model integrates five complementary theoretical perspectives around a common intention–behavior pathway. The integration is based on a division of theoretical roles rather than on the assumption that the five perspectives constitute one unified theory. The Privacy Paradox framework, Technostress Theory, Affect Theory, and Diffusion of Innovation Theory explain four distinct sources of influence that may shape students’ willingness to use ChatGPT: perceived privacy-related risk, technology-related psychological discomfort, affective responses, and individual innovativeness, respectively. The Theory of Planned Behavior provides the overarching behavioral mechanism by conceptualizing behavioral intention as the proximal motivational state through which these antecedent influences may be associated with subsequent technology-use behavior.

This structure is particularly relevant to generative AI because adoption may involve both facilitating and inhibiting influences. Students may simultaneously perceive ChatGPT as an innovative and useful learning technology while experiencing privacy concerns, technology-related discomfort, or guilt regarding its academic use. The proposed framework therefore does not assume that these influences operate through identical psychological processes. Instead, it proposes that their effects converge at the level of behavioral intention, which represents students’ readiness or willingness to engage with ChatGPT, before being reflected in self-reported usage behavior. Consequently, the model permits the relative contribution of different theoretical perspectives to be examined within the same intention–behavior framework. Drawing on the Privacy Paradox framework, Technostress Theory, Affect Theory, Diffusion of Innovation Theory, and the Theory of Planned Behavior, the study examines how psychological barriers and individual characteristics are associated with undergraduate medical students’ behavioral intention to use ChatGPT and how behavioral intention is subsequently associated with self-reported ChatGPT usage behavior.

#### Privacy concern and behavioral intention

Privacy concern refers to individuals’ perceptions of the potential risks associated with the collection, storage, processing, and use of personal information in digital environments (37). Because generative AI systems require users to enter information and generate responses based on those inputs, concerns about data confidentiality, information handling, and potential secondary use of information may influence students’ willingness to engage with AI-assisted learning technologies. In the present study, privacy concern is conceptualized from a privacy-risk perspective rather than as an expression of the privacy paradox. The privacy paradox specifically refers to a discrepancy between individuals’ stated privacy concerns and their subsequent disclosure or privacy-related behavior (29). Because the present study does not directly examine such a discrepancy, the privacy paradox is not used as the primary theoretical explanation for H1. Instead, a privacy-risk perspective provides a more direct rationale for examining whether perceived privacy-related risks are associated with students’ behavioral intention to use ChatGPT.

Previous studies have reported that higher levels of privacy concern can be associated with lower trust and weaker behavioral intentions across digital and technology-mediated contexts (37, 38). In the context of generative AI, concerns regarding data confidentiality and uncertainty about how user-provided information may be processed or used may similarly increase perceived risk and reduce willingness to engage with AI systems. These concerns may be particularly relevant in medical education, where students may interact with academic information and clinically related learning materials and are expected to maintain high standards of confidentiality. Thus, privacy concern represents a potential inhibiting influence on behavioral intention rather than a manifestation of a privacy paradox. Students who perceive greater privacy-related risks associated with ChatGPT may be less willing to incorporate the technology into their academic activities. Accordingly, students reporting higher levels of privacy concern are expected to report lower behavioral intentions to use ChatGPT.

**H1:** Privacy concern is negatively associated with behavioral intention to use ChatGPT.

#### Technophobia and behavioral intention

Technophobia refers to feelings of fear, anxiety, or discomfort experienced when interacting with unfamiliar or complex technologies (20). Although technophobia is related to broader forms of technology-related psychological strain, it should not be treated as synonymous with technostress. Technostress generally concerns psychological strain arising from technology-related demands, such as complexity, overload, invasion, insecurity, and uncertainty (30), whereas technophobia focuses more specifically on an individual's affective apprehension, anxiety, or discomfort toward technology (20, 21).

The relationship between technophobia and behavioral intention can be understood through Technostress Theory, which suggests that rapid technological change may be accompanied by perceptions of complexity, uncertainty, and reduced control, resulting in technology-related psychological strain (30). Students reporting higher levels of technophobia may perceive ChatGPT as difficult to use, cognitively demanding, or less reliable, which may be associated with lower willingness to incorporate the technology into their learning activities. Previous studies have consistently reported negative associations between technology-related anxiety and behavioral intention across a range of digital technologies (21, 39, 40). Thus, the present study does not assume that technophobia is a direct manifestation or dimension of technostress. Rather, Technostress Theory provides a broader explanatory context for technology-related psychological strain, while research specifically addressing technophobia and technology anxiety provides the direct conceptual basis for examining technophobia as a psychological barrier to ChatGPT adoption. In the context of medical education, where AI-assisted learning is becoming increasingly prevalent, technophobia may therefore represent an important factor associated with students’ acceptance of ChatGPT. Accordingly, students reporting higher levels of technophobia are expected to report lower behavioral intentions to use ChatGPT.

**H2:** Technophobia is negatively associated with behavioral intention to use ChatGPT.

#### Guilt feelings and behavioral intention

Guilt feelings refer to the self-conscious emotional experience that arises when individuals perceive their actions as conflicting with their personal values, moral standards, or socially accepted norms (31). In the context of generative AI use in education, guilt may arise when students perceive their use of ChatGPT as inconsistent with expectations concerning academic integrity, independent learning, originality, or appropriate use of technological assistance.

In the present study, guilt is conceptualized specifically as experienced academic-integrity-related guilt associated with recent ChatGPT use. This conceptualization is based on the temporal orientation of the questionnaire, which asks students to reflect on guilt experienced during their recent use of ChatGPT. Accordingly, the construct is not treated as purely anticipated guilt, which would refer to an expectation of feeling guilty before engaging in a future behavior. Rather, it captures students’ experienced emotional response to their recent ChatGPT use. This relationship can be understood through cognitive appraisal theory (31). Cognitive appraisal theory proposes that individuals evaluate situations and their own behavior in relation to their goals, values, and perceived standards, and that these appraisals can generate emotional responses that subsequently influence behavioral decisions. Applied to ChatGPT use, students who appraise their recent use as inconsistent with academic-integrity expectations or their desired level of independent learning may experience stronger guilt. Previous studies have found that guilt feelings prevent people from behaviors violating the moral or social norms (22, 41, 42). The proposed relationship also involves a temporal distinction between experienced emotion and subsequent behavioral intention. Although guilt is assessed retrospectively in relation to recent ChatGPT use, behavioral intention concerns students’ willingness to use ChatGPT in the future. Thus, the proposed mechanism is that students’ appraisal of their recent ChatGPT use generates an experienced moral-affective response, and this response may subsequently influence their willingness to continue using the technology. Because guilt signals a perceived discrepancy between one's behavior and valued standards, students experiencing stronger ChatGPT-related guilt may be less willing to continue using ChatGPT for academic purposes. Accordingly, guilt is treated in the present study as an experienced academic-integrity-related moral emotion arising from recent ChatGPT use that may influence subsequent behavioral intention, rather than as a generic affective reaction or a direct measure of anticipated guilt.

**H3:** Guilt feelings are negatively associated with behavioral intention to use ChatGPT.

#### Personal innovativeness and behavioral intention

Personal innovativeness reflects an individual's willingness to experiment with and adopt new technologies earlier than others (23). Individuals with higher levels of personal innovativeness are generally more willing to embrace technological change and experiment with emerging digital tools.

This relationship is explained by Diffusion of Innovation Theory, which proposes that innovative individuals are usually more tolerant of uncertainty, aware of the benefits of new technology, and faster in adopting innovations in comparison with the rest of the population (32). In the context of generative AI, people with high personal innovativeness can see ChatGPT as an educational asset that can be incorporated into their learning strategy and experimented with. Previous studies consistently identify personal innovativeness as a positive predictor of technology adoption across educational and digital contexts (23, 32–35). Considering the novelty of generative AI, it is therefore expected that innovative medical students will have greater intentions of using ChatGPT. Accordingly, higher levels of personal innovativeness are expected to be associated with stronger behavioral intention to use ChatGPT.

**H4:** Personal innovativeness is positively associated with behavioral intention to use ChatGPT.

#### Behavioral intention and ChatGPT usage behavior

Behavioral intention reflects an individual's readiness to perform a particular behavior and is widely regarded as a proximal predictor of subsequent behavior (24). In the present study, however, usage behavior was measured through self-reported questionnaire items rather than objectively recorded platform activity. Therefore, the hypothesized relationship concerns behavioral intention and students’ reported engagement with ChatGPT rather than objectively verified usage. In the context of ChatGPT, behavioral intention reflects students’ willingness to integrate the technology into their academic activities, including studying, academic writing, examination preparation, and problem-solving.

The present study draws on the intention–behavior component of the Theory of Planned Behavior (TPB), which conceptualizes behavioral intention as a proximal motivational construct associated with subsequent behavior (24). Importantly, this use of TPB is limited to the intention–behavior linkage; the canonical TPB constructs of attitude, subjective norm, and perceived behavioral control were not included in the present model. Thus, the present study does not constitute a full test of TPB. This relationship is particularly relevant because ChatGPT remains a voluntary educational technology, allowing students to decide whether and how frequently they incorporate it into their learning activities. Previous research consistently demonstrates that stronger behavioral intentions are associated with greater technology use across a wide range of educational and digital contexts (24, 25, 34, 35).

**H5:** Behavioral intention is positively associated with ChatGPT usage behavior.

#### Behavioral intention as a mediator

The Theory of Planned Behavior suggests that psychological and individual factors may influence subsequent behavior through behavioral intention, which represents a proximal determinant of behavior (24). In the case of generative AI, before making decisions regarding the technology, students assess it, develop the willingness to use the technology, and then make decisions about adopting it to their learning activities. Therefore, behavioral intention is conceptualized as the mediating mechanism through which privacy concern, technophobia, guilt feelings, and personal innovativeness may be associated with self-reported ChatGPT usage behavior.

Students reporting greater privacy concern, technophobia, or guilt feelings are expected to develop weaker intentions to use ChatGPT, whereas students with higher levels of personal innovativeness are expected to develop stronger behavioral intentions. Consistent with previous technology adoption research, behavioral intention is therefore expected to mediate the relationships between these antecedent variables and self-reported ChatGPT usage behavior (24, 25, 43). Thus, considering generative AI, behavioral intention is also assumed to mediate the relationships between the aforementioned antecedents and the target behavior. Therefore, the following hypotheses are suggested:

**H6:** Behavioral intention mediates the relationship between privacy concern and ChatGPT usage behavior.

**H7:** Behavioral intention mediates the relationship between technophobia and ChatGPT usage behavior.

**H8:** Behavioral intention mediates the relationship between guilt feelings and ChatGPT usage behavior.

**H9:** Behavioral intention mediates the relationship between personal innovativeness and ChatGPT usage behavior.

## Methods

### Study design and participants

This study employed a cross-sectional quantitative survey design to investigate the psychological and individual factors associated with ChatGPT adoption among undergraduate medical students. The study was conducted between February and April 2026 at King Faisal University, located in the Eastern Province of Saudi Arabia. Although the study was conducted at a single institution, King Faisal University attracts students from diverse geographical regions across Saudi Arabia, resulting in a geographically and culturally diverse study population. The target population comprised undergraduate medical students enrolled in the MBBS program. Participants were recruited using convenience sampling through an anonymous, self-administered online questionnaire. Ethical approval was obtained from the Deanship of Scientific Research, King Faisal University under Approval No. KFU-REC-2025-OCT-ETHICS3617 dated 09 October 2025. All study procedures were conducted in accordance with the ethical principles of the Declaration of Helsinki and other relevant ethical guidelines governing research involving human participants (44). Before accessing the questionnaire, participants were provided with information regarding the study objectives, procedures, voluntary participation, confidentiality, and their right to withdraw at any time without penalty. Electronic informed consent was obtained from all participants before completion of the questionnaire. No personally identifiable information was collected, and all responses were stored anonymously and confidentially.

### Sample size calculation

Sample size adequacy was evaluated in accordance with current recommendations for Partial Least Squares Structural Equation Modeling (PLS-SEM), which emphasize statistical power and model complexity (45). *A priori* power analysis was conducted using G*Power 3.1.9.7. Assuming a medium effect size (f^2^ = 0.15), a significance level of *α* = .05, statistical power of.95, and four predictors for the endogenous construct with the largest number of incoming structural paths (behavioral intention), the minimum required sample size was 129 participants. A total of 350 undergraduate medical students were invited to participate, of whom 243 completed the questionnaire, yielding a response rate of 69.4%. As the achieved sample size substantially exceeded the minimum requirement, it was considered adequate for support estimation of the measurement and structural models, and testing the hypothesized relationships (46, 47).

The final sample of 243 comprised 124 male students (51.1%) and 119 female students (48.9%), with ages ranging from 19 to 25 years (*M* = 21.6, *SD* = 1.8). Participants represented all academic levels of the MBBS program, with 16.0% enrolled in Year 1, 13.6% in Year 2, 27.6% in Year 3, 17.7% in Year 4, 14.0% in Year 5, and 11.1% undertaking their internship year. Regarding academic performance, 6.2% of participants reported a cumulative GPA between 2.0 and 2.99, 20.6% between 3.0 and 3.99, 35.5% between 4.0 and 4.49, and 40.7% between 4.5 and 5.0, whereas none of the participants reported a GPA below 2.0. Regarding family structure, 51.0% of participants belonged to nuclear families and 49.0% to joint families. Most respondents (82.7%) resided in urban areas, whereas 17.3% lived in rural areas. Monthly household income was reported as less than SAR 5,000 by 4.1% of participants, SAR 5,001–10,000 by 16.5%, SAR 10,001–15,000 by 26.0%, and more than SAR 15,000 by 52.7%.

### Data collection tool

Data were collected using a structured, self-administered online questionnaire designed to assess the psychological and individual factors associated with ChatGPT adoption among undergraduate medical students. The questionnaire was developed following a comprehensive review of the relevant literature, with measurement items adapted from previously validated instruments to ensure content validity, construct validity, and contextual relevance (45, 48). The adaptation involved contextual wording modifications to align the original items specifically with ChatGPT use and the undergraduate medical-education setting, while preserving their original conceptual meaning, dimensional structure, and reflective nature. No new substantive indicators were introduced during the adaptation process. For the guilt construct specifically, the three items were adapted from Masood et al. (2022) (22), whose original measure assessed social-media-related guilt. The behavioral referent was contextualized to recent ChatGPT use, while the original emotional content and response structure were retained. The descriptors “guilty,” “ashamed,” and “blameworthy” were retained because they represent related aspects of self-conscious moral-affective evaluation associated with one's behavior. The adapted questionnaire was subsequently pilot-tested among 30 undergraduate medical students to assess clarity, comprehensibility, and contextual relevance. Particular attention was given to whether participants understood the ChatGPT-specific behavioral referent of the adapted items. Minor wording refinements were made based on the pilot participants’ feedback, while the pilot data were excluded from the final analysis. Because the guilt items referred to participants’ recent experience with ChatGPT, guilt was operationalized as experienced academic-integrity-related guilt associated with recent use rather than anticipated guilt regarding future use. Participants were instructed to respond to these items with reference to their recent ChatGPT experience. The questionnaire consisted of two sections. The first section collected participants’ demographic and academic information, including age, sex, academic year, cumulative GPA, family structure, area of residence, monthly household income, and accommodation status. The second section assessed the six latent constructs included in the conceptual framework: privacy concern, technophobia, guilt feelings, personal innovativeness, behavioral intention, and ChatGPT usage behavior. Privacy concern was measured using items adapted from Brill et al. (2019) (49). Technophobia was measured using the instrument developed by Khasawneh (2018) (21). Guilt feelings were measured using the scale proposed by Masood et al. (2022) (22). Personal innovativeness was measured using the Personal Innovativeness in Information Technology (PIIT) scale developed by Agarwal and Prasad (1998) (23). Behavioral intention was measured using items adapted from the UTAUT2 instrument developed by Venkatesh et al. (2012) (25); and represented students’ willingness or intention to use ChatGPT for educational purposes. ChatGPT usage behavior was measured using items adapted from McLean and Osei-Frimpong (2019) (50) and Aparicio et al. (2017) (51) and represented students’ self-reported engagement with ChatGPT. Thus, behavioral intention and usage behavior were operationalized as conceptually distinct constructs, with the former reflecting intended future use and the latter reflecting reported engagement with the technology. Because usage behavior was assessed through self-report rather than objective platform-use records, it is referred to throughout the manuscript as self-reported ChatGPT usage behavior. The questionnaire comprised 17 measurement items, with each latent construct operationalized through reflective indicators derived from established measurement instruments. A reflective measurement specification was adopted because the indicators were conceptualized as manifestations of their respective latent constructs and were therefore expected to be highly intercorrelated. The reflective specification was retained from the conceptualization of the source instruments and was not altered as part of the contextual adaptation. The questionnaire was administered in English, which is the official language of instruction for the MBBS program at King Faisal University. All questionnaire items were measured using a seven-point Likert scale ranging from 1 (strongly disagree) to 7 (strongly agree). A seven-point response format was selected because it provides greater response discrimination, enhances measurement precision, and has been shown to improve the psychometric properties of measurement scales compared with response formats containing fewer categories (45). The complete list of constructs, corresponding measurement items, and their original sources is presented in Appendix A (Table A1).

### Procedure

Following approval from the institutional review board, the recruitment process for the participants was undertaken by using student representatives for each academic year. A total of 350 eligible undergraduate medical students were invited to participate. The invitation letter, participant information form, and a link to the online survey were sent to the designated WhatsApp groups of the university with the permission of respective group admins. The 350-student denominator was based on the number of eligible students reached through these designated recruitment channels. The participants were briefed about the objective of the study, data collection procedure, and that their participation is voluntary along with the steps taken by the researchers to maintain their confidentiality and anonymity. Consent of the participants was taken electronically prior to starting the survey. Throughout the data collection period, trained senior medical students served as facilitators, addressing participants’ queries and providing support when needed. Prior to the main survey, a pilot study involving 30 undergraduate medical students was conducted to evaluate the clarity, comprehensibility, and relevance of the questionnaire items. Minor wording refinements were made based on participants’ suggestions, without changing the underlying constructs or response format. Pilot test data were excluded from the final data analysis. The questionnaire was administered in English, which is the official language of instruction for the MBBS program at King Faisal University. Therefore, translation and back-translation were not required. No language-equivalence difficulties were identified during pilot testing. The pilot was not intended to establish psychometric validity; rather, it was used to assess the clarity and contextual interpretation of the adapted items before administration of the main survey. Evidence regarding reliability and construct validity was subsequently examined using the final study sample through indicator reliability, internal consistency reliability, convergent validity, and discriminant validity analyses.

### Statistical analysis

Data were analysed using the Statistical Package for the Social Sciences (SPSS) version 27 (IBM Corp., Armonk, NY, USA) for descriptive statistical analyses and SmartPLS 4.1s (SmartPLS GmbH, Oststeinbek, Germany) for Partial Least Squares Structural Equation Modelling (PLS-SEM). Descriptive statistics, including frequencies, percentages, means, and standard deviations, were computed to summarize participants’ demographic and academic characteristics.

PLS-SEM was employed to test the proposed conceptual model because the study focused on examining predictive and explanatory relationships among multiple latent constructs and assessing behavioral intention as a mediating mechanism linking psychological and individual antecedents with self-reported ChatGPT usage behavior. Although the theoretically specified and confirmatory nature of the present model, the use of established reflective measurement scales, and the achieved sample size would also make covariance-based structural equation modelling (CB-SEM) a viable analytical approach, PLS-SEM was selected because the study had a combined explanatory and predictive objective, with particular emphasis on explaining variance in Behavioral Intention and self-reported ChatGPT usage behavior and estimating the indirect associations within the proposed model. The choice of PLS-SEM was therefore not based solely on sample-size considerations, the reflective nature of the constructs, or the number of latent variables, but primarily on the study's emphasis on explained variance and prediction within an integrated latent-variable model. PLS-SEM is a variance-based structural equation modelling technique that permits simultaneous evaluation of the measurement and structural models and is appropriate for models involving multiple latent variables and indirect effects (45). Accordingly, PLS-SEM was used as the primary estimation approach while recognizing that CB-SEM represents a suitable alternative for confirmatory assessment of the theoretically specified model. The bootstrapping procedure available in PLS-SEM was also used to estimate the significance and confidence intervals of the hypothesized direct and indirect effects. The measurement model was evaluated by examining indicator reliability, internal consistency reliability, convergent validity, and discriminant validity. Indicator reliability was assessed using standardized outer loadings, with values of 0.70 or higher considered (45). Internal consistency reliability was assessed using Cronbach's alpha (CA) and composite reliability (CR), with values between 0.70 and 0.95 considered acceptable (45). Convergent validity was evaluated using the average variance extracted (AVE), with values greater than 0.50 indicating that a construct explained more than half of the variance of its indicators (52). Discriminant validity was assessed using both the Fornell–Larcker criterion and the heterotrait–monotrait ratio (HTMT). According to the Fornell–Larcker criterion, the square root of the AVE for each construct should exceed its correlations with other constructs, whereas HTMT values below 0.90 were considered indicative of satisfactory discriminant validity (53).

The structural model was subsequently evaluated by examining collinearity, explanatory power, effect size, predictive relevance, and the significance of the hypothesized relationships. Multicollinearity among predictor constructs was assessed using variance inflation factor (VIF) values, with values below 5.0 indicating that multicollinearity was not a concern (45). The explanatory power of the model was evaluated using the coefficient of determination (R^2^), whereas the contribution of individual exogenous constructs was assessed using effect size (f^2^) following the guidelines proposed by Cohen (1988) (54). Predictive relevance was initially examined using the Stone–Geisser predictive relevance (Q^2^) statistic, with values greater than zero indicating in-sample predictive relevance (45). Because Q^2^ does not constitute an out-of-sample predictive assessment, PLSpredict was additionally conducted to evaluate the model's predictive performance on observations not used in model estimation. The PLSpredict results were interpreted by comparing the prediction errors of the PLS-SEM model with those of the linear-model benchmark. The statistical significance of the hypothesized direct and indirect relationships was evaluated using the bootstrapping procedure with 5,000 resamples, generating standardized path coefficients (*β*), standard errors, *t*-values, *p*-values, and bias-corrected 95% bootstrap confidence intervals (BCa CIs). Mediation was assessed by examining the significance of the specific indirect effects following the recommendations of Zhao et al. (2010) (55). Because the study employed a cross-sectional design, mediation findings were interpreted as statistical indirect associations consistent with the hypothesized model rather than evidence of temporal or causal mediation. The standardized root mean square residual (SRMR) was reported as a supplementary model-quality indicator of standardized residual discrepancy, with lower values indicating smaller discrepancies between the observed and model-implied correlations. An SRMR value below 0.08 was considered indicative of relatively low residual discrepancy (45). However, SRMR was not treated as definitive evidence of overall model fit, and model evaluation primarily emphasized measurement quality, discriminant validity, collinearity, explained variance (R^2^), effect sizes (f^2^), predictive relevance (Q^2^), path significance, and out-of-sample predictive performance assessed using PLSpredict. Because all study variables were collected using self-administered questionnaires from the same respondents within a single cross-sectional survey, the possibility of common method bias (CMB) was considered. Harman's single-factor test was initially used as a preliminary diagnostic. A first-factor variance below 50% was interpreted as indicating that no single factor accounted for the majority of the observed variance; however, Harman's single-factor test alone cannot establish the absence of common method variance. Therefore, as an additional diagnostic, full-collinearity variance inflation factor (VIF) values were examined in the PLS-SEM analysis. Following the recommended approach, VIF values below 3.3 were considered to provide evidence against severe common method bias. The Harman's single-factor test and full-collinearity VIF assessment were interpreted as complementary diagnostics rather than definitive proof that common method variance was absent. Finally, to evaluate the robustness of the proposed structural model, an additional PLS-SEM analysis was conducted by adjusting for age, gender, academic year, cumulative GPA, and area of residence as demographic and academic control variables.

## Results

### Measurement model assessment

The measurement model was evaluated using Partial Least Squares Structural Equation Modelling (PLS-SEM) by assessing internal consistency reliability, convergent validity, indicator reliability, discriminant validity, and overall model fit. Internal consistency reliability was examined using Cronbach's alpha (CA) and composite reliability (CR), whereas convergent validity was assessed using the average variance extracted (AVE).

As presented in Table 1, all constructs demonstrated satisfactory internal consistency. Cronbach's alpha values ranged from 0.784 to 0.911, while composite reliability values ranged from 0.861 to 0.945, exceeding the recommended threshold of 0.70 and remaining below the upper limit of 0.95, thereby indicating good reliability (56). Likewise, all AVE values exceeded the recommended threshold of 0.50 (range = 0.675–0.896), confirming satisfactory convergent validity (45).

**Table 1:** Result of reliability and validity tests.

Constructs/ Items	Factor loading					
	BI	CUB	GF	IV	PC	TP
Behavioral intention (BI): CA = 0.821, CR = 0.894, AVE = 0.737						
BI1	0.841					
BI2	0.837					
BI3	0.897					
ChatGPT Usage Behavior (CUB); CA = 0.884, CR = 0.945, AVE = 0.896						
CUB1		0.945				
CUB2		0.948				
Guilt Feeling (GF): CA = 0.911, CR = 0.943, AVE = 0.847						
GF1			0.937			
GF2			0.912			
GF3			0.911			
Innovativeness (IV): CA = 0.900, CR = 0.938, AVE = 0.835						
IV1				0.864		
IV2				0.927		
IV3				0.948		
Privacy Concern (PC): CA = 0.822, CR = 0.894, AVE = 0.737						
PC1					0.866	
PC2					0.866	
PC3					0.843	
Technophobia (TP): CA = 0.784, CR = 0.861, AVE = 0.675						
TP1						0.894
TP2						0.797
TP3						0.769

CA, Cronbach's alpha, CR, composite reliability; AVE, average variance extracted.

Indicator reliability was subsequently assessed by examining the standardized outer loadings of the measurement items. As shown in Table 1, all indicators loaded strongly on their respective constructs, with standardized loadings exceeding the recommended threshold of 0.70 (57). These findings indicate that each indicator adequately represented its intended latent construct and further support the reliability of the measurement model.

Discriminant validity was evaluated using both the Fornell–Larcker criterion and the heterotrait–monotrait ratio (HTMT). As shown in Table 2, the square root of the AVE for each construct exceeded its correlations with all other constructs, satisfying the Fornell–Larcker criterion and indicating adequate discriminant validity. Most HTMT values were below the recommended threshold of 0.90. However, the HTMT value between Behavioral Intention and ChatGPT Usage Behavior was 0.912, indicating some empirical proximity between these two conceptually related constructs. This finding was interpreted cautiously rather than as unequivocal evidence of complete discriminant validity. Nevertheless, the two constructs were retained separately because they represent conceptually distinct stages of the proposed adoption process: Behavioral Intention reflects students’ intention or readiness to use ChatGPT, whereas ChatGPT Usage Behavior reflects their self-reported engagement with the technology. Thus, although the slightly elevated HTMT value warrants caution in interpreting the distinction between these constructs, the Fornell–Larcker criterion and the remaining HTMT values provided supportive evidence for the overall distinctiveness of the measurement constructs.

**Table 2:** Discriminant validity assessment using the fornell–larcker criterion and heterotrait–monotrait (HTMT) ratio.

Construct	BI	CUB	GF	IV	PC	TP
Behavioral Intention (BI)	**0** **.** **859**	0.912	0.084	0.712	0.432	0.260
ChatGPT Usage Behavior (CUB)	0.777	**0**.**946**	0.028	0.606	0.402	0.269
Guilt Feeling (GF)	−0.075	0.012	**0**.**920**	0.054	0.138	0.310
Innovativeness (IV)	0.615	0.543	0.047	**0**.**914**	0.562	0.449
Privacy Concern (PC)	0.356	0.341	0.113	0.486	**0**.**858**	0.720
Technophobia (TP)	0.236	0.240	0.250	0.386	0.595	**0**.**822**

The bold diagonal values represent the square root of the Average Variance Extracted (AVE) (Fornell–Larcker criterion). Values below the diagonal are the latent variable correlations (Fornell–Larcker), whereas values above the diagonal are the Heterotrait–Monotrait ratio (HTMT).

To further evaluate the adequacy of the measurement model, overall model fit was assessed using the standardized root mean square residual (SRMR). The obtained SRMR value of 0.056 was below the recommended threshold of 0.08, indicating an acceptable model fit and suggesting that the proposed measurement model adequately reproduced the observed covariance matrix (45)

### Structural model analysis

Following confirmation of the adequacy of the measurement model, the structural model was evaluated using the bootstrapping procedure implemented in SmartPLS with 5,000 bootstrap resamples. Standardized path coefficients (*β*), bias-corrected 95% bootstrap confidence intervals (BC CIs), t-values, and *p*-values were estimated using two-tailed significance testing to evaluate the proposed hypotheses. The results of the structural model are presented in Figure 2 and Table 3. The findings revealed that only two direct relationships were statistically significant. Innovativeness exerted a significant positive effect on Behavioral Intention (*β* = 0.579, *t* = 11.007, *p* < 0.001), thereby supporting H4. Likewise, Behavioral Intention had a strong positive effect on ChatGPT Usage Behavior (*β* = 0.777, *t* = 24.390, *p* < 0.001), providing support for H5. In contrast, Privacy Concern did not significantly predict Behavioral Intention (*β* = 0.099, *t* = 1.309, *p* = 0.191), resulting in the rejection of H1. Notably, the direction of this structural coefficient was positive, rather than the negative direction hypothesized in H1. This positive direction was also observed in the bivariate association between Privacy Concern and Behavioral Intention reported in Table 2 (r = 0.356), although the structural coefficient was considerably smaller and statistically non-significant after the other predictors were considered simultaneously. Similarly, Technophobia showed no statistically significant relationship with Behavioral Intention (*β* = −0.020, *t* = 0.308, *p* = 0.758), while Guilt Feelings also failed to significantly predict Behavioral Intention (*β* = −0.109, *t* = 1.796, *p* = 0.073). For Technophobia, the bivariate correlation with Behavioral Intention was positive (r = 0.236), whereas the structural coefficient became slightly negative (*β* = −0.020). This sign reversal indicates that the positive zero-order association was not retained after the other predictors were included in the structural model and may reflect shared variance or overlap among the predictor constructs. Because a formal suppression analysis was not conducted, this pattern was interpreted cautiously rather than being classified definitively as a suppression effect. Consequently, H2 and H3 were not supported.

**Figure 2:**
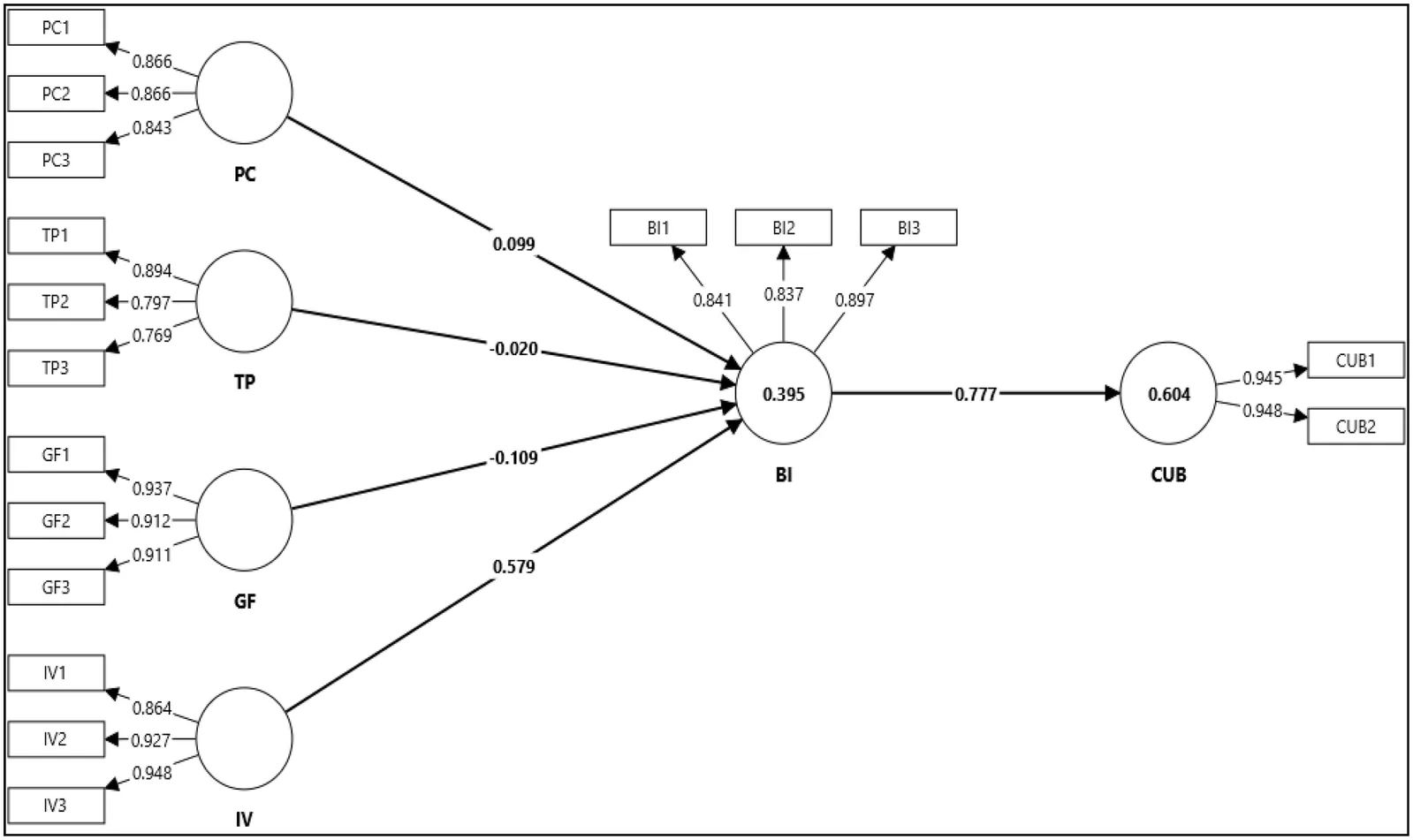
Structural model with standardized path coefficients.

**Table 3:** Results of hypotheses testing.

Hypothesis	Path Relationship	Effect	*β*	SE	*t*	*p*	95% BC CI	Support
H1	Privacy Concern → Behavioral Intention	Direct	0.099	0.076	1.309	.191	(-0.049, 0.250)	Not Supported
H2	Technophobia → Behavioral Intention	Direct	-0.020	0.065	0.308	.758	(-0.166, 0.093)	Not Supported
H3	Guilt feelings → Behavioral Intention	Direct	-0.109	0.061	1.796	.073	(-0.195, 0.080)	Not Supported
H4	Innovativeness → Behavioral Intention	Direct	0.579	0.053	11.007	<.001	(0.476, 0.682)	Supported
H5	Behavioral Intention → ChatGPT Usage Behavior	Direct	0.777	0.032	24.390	<.001	(0.709, 0.835)	Supported
H6	Privacy Concern → Behavioral Intention → ChatGPT Usage Behavior	Indirect	0.077	0.059	1.309	.190	(-0.038, 0.194)	Not Supported
H7	Technophobia → Behavioral Intention → ChatGPT Usage Behavior	Indirect	-0.016	0.053	0.309	.758	(-0.129, 0.072)	Not Supported
H8	Guilt feelings → Behavioral Intention → ChatGPT Usage Behavior	Indirect	-0.085	0.047	1.818	.069	(-0.149, 0.060)	Not Supported
H9	Innovativeness → Behavioral Intention → ChatGPT Usage Behavior	Indirect	0.450	0.050	8.949	<.001	(0.357, 0.554)	Supported

*Β*, standardized path coefficient; BC CI, bias-corrected 95% bootstrap confidence interval based on 5,000 bootstrap resamples.

The hypothesized indirect associations through Behavioral Intention were subsequently examined using the bootstrapped specific indirect effects. A significant indirect association was observed for Innovativeness → Behavioral Intention → ChatGPT Usage Behavior (*β* = 0.450, t = 8.949, *p* < 0.001, 95% BC CI [0.357, 0.554]), supporting H9. This finding indicates that Innovativeness was significantly associated with ChatGPT Usage Behavior through Behavioral Intention. However, because the present structural model did not estimate direct paths from Innovativeness to ChatGPT Usage Behavior, this finding is interpreted as a significant specific indirect effect rather than being classified into a conventional mediation category. The specific indirect effects through Behavioral Intention were not statistically significant for Privacy Concern (*β* = 0.077, *p* = .190, 95% BC CI [−0.038, 0.194]), Technophobia (*β* = −0.016, *p* = .758, 95% BC CI [−0.129, 0.072]), or Guilt Feelings (*β* = −0.085, *p* = .069, 95% BC CI [−0.149, 0.060]). Accordingly, H6, H7, and H8 were not supported. These findings are interpreted as non-significant specific indirect effects rather than as evidence of the absence of conventional mediation, because the corresponding direct antecedent → ChatGPT Usage Behavior paths were not estimated.

Overall, the structural model indicates that Innovativeness was the only significant antecedent of Behavioral Intention, whereas Behavioral Intention emerged as the principal determinant of ChatGPT Usage Behavior. None of the examined psychological barriers—Privacy Concern, Technophobia, and Guilt Feelings—demonstrated statistically significant direct or indirect effects on ChatGPT adoption.

To further examine the robustness of the proposed structural model, an additional PLS-SEM analysis was conducted by including age, gender, academic year, cumulative GPA, and area of residence as control variables predicting ChatGPT Usage Behavior. The adjusted structural results are reported in Table 4. The inclusion of these controls did not change the direction or statistical significance pattern of the hypothesized relationships. Specifically, Privacy Concern → Behavioral Intention remained non-significant (*β* = 0.012, SE = 0.074, t = 0.165, *p* = .869, 95% BCa CI [−0.128, 0.163]); Technophobia → Behavioral Intention remained non-significant (*β* = −0.039, SE = 0.063, t = 0.622, *p* = .534, 95% BCa CI [−0.180, 0.073]); and Guilt Feelings → Behavioral Intention remained non-significant (*β* = −0.056, SE = 0.061, t = 0.914, *p* = .361, 95% BCa CI [−0.138, 0.122]). In contrast, Innovativeness continued to significantly predict Behavioral Intention (*β* = 0.708, SE = 0.047, t = 15.023, *p* < .001, 95% BCa CI [0.614, 0.798]), and Behavioral Intention remained a strong predictor of ChatGPT Usage Behavior (*β* = 0.851, SE = 0.023, t = 37.831, *p* < .001, 95% BCa CI [0.803, 0.891]). The control variables themselves were not significant predictors of ChatGPT Usage Behavior. Age (*β* = 0.043, SE = 0.073, t = 0.593, *p* = .553, 95% BCa CI [−0.104, 0.184]), gender (*β* = −0.092, SE = 0.072, t = 1.280, *p* = .201, 95% BCa CI [−0.232, 0.048]), academic year (*β* = −0.069, SE = 0.074, t = 0.932, *p* = .352, 95% BCa CI [−0.212, 0.077]), GPA (*β* = 0.004, SE = 0.035, t = 0.100, *p* = .920, 95% BCa CI [−0.065, 0.075]), and area of residence (*β* = −0.044, SE = 0.099, t = 0.446, *p* = .656, 95% BCa CI [−0.238, 0.156]) were all non-significant. Thus, although the magnitude of some substantive path coefficients changed after adjustment, the overall pattern of supported and unsupported hypotheses remained unchanged. Behavioral Intention continued to mediate only the relationship between Innovativeness and ChatGPT Usage Behavior.

**Table 4:** Structural model results and robustness analysis (bootstrapping = 5,000).

Hypothesis	Structural Path	β	SE	*t*	*p*	95% BCa CI	Decision
Direct effects							
H1	Privacy Concern → Behavioral Intention	0.012	0.074	0.165	.869	[-0.128, 0.163]	Not supported
H2	Technophobia → Behavioral Intention	-0.039	0.063	0.622	.534	[-0.180, 0.073]	Not supported
H3	Guilt feelings → Behavioral Intention	-0.056	0.061	0.914	.361	[-0.138, 0.122]	Not supported
H4	Innovativeness → Behavioral Intention	0.708	0.047	15.023	<.001	[0.614, 0.798]	Supported
H5	Behavioral Intention → ChatGPT Usage Behavior	0.851	0.023	37.831	<.001	[0.803, 0.891]	Supported
Indirect effects (Mediation)							
H6	Privacy Concern → Behavioral Intention → ChatGPT Usage Behavior	0.010	0.063	0.165	.869	[-0.109, 0.139]	Not supported
H7	Technophobia → Behavioral Intention → ChatGPT Usage Behavior	-0.034	0.054	0.623	.533	[-0.151, 0.063]	Not supported
H8	Guilt feelings → Behavioral Intention → ChatGPT Usage Behavior	-0.047	0.052	0.917	.359	[-0.117, 0.101]	Not supported
H9	Innovativeness → Behavioral Intention → ChatGPT Usage Behavior	0.603	0.048	12.636	<.001	[0.509, 0.697]	Supported
Control variables							
—	Age → ChatGPT Usage Behavior	0.043	0.073	0.593	.553	[-0.104, 0.184]	Not significant
—	Gender → ChatGPT Usage Behavior	-0.092	0.072	1.280	.201	[-0.232, 0.048]	Not significant
—	Academic Year → ChatGPT Usage Behavior	-0.069	0.074	0.932	.352	[-0.212, 0.077]	Not significant
—	GPA → ChatGPT Usage Behavior	0.004	0.035	0.100	.920	[-0.065, 0.075]	Not significant
—	Area of Residence (Urban/Rural) → ChatGPT Usage Behavior	-0.044	0.099	0.446	.656	[-0.238, 0.156]	Not significant

*Β*, standardized path coefficient; SE, bootstrap standard error; BCa CI, bias-corrected and accelerated 95% bootstrap confidence interval. Structural relationships were evaluated using bootstrapping with 5,000 resamples.

Behavioral Intention was not treated as a control-variable outcome in this robustness analysis because it was specified *a priori* as the theoretically central endogenous mediator linking the antecedent psychological and individual factors to ChatGPT Usage Behavior. The demographic and academic variables were therefore introduced as background covariates specifically to examine whether the observed usage relationship remained after accounting for potentially relevant individual characteristics. Accordingly, the present robustness analysis evaluated control-variable effects on ChatGPT Usage Behavior rather than treating Behavioral Intention itself as a covariate.

### Structural model quality assessment

The quality of the structural model was further evaluated by examining its explanatory power (R^2^), effect sizes (f^2^), predictive relevance (Q^2^), multicollinearity using variance inflation factors (VIF), and common method bias. The results are presented in Table 5. The proposed model explained 39.5% of the variance in Behavioral Intention (R^2^ = 0.395), indicating moderate explanatory power, and 60.4% of the variance in ChatGPT Usage Behavior (R^2^ = 0.604), indicating substantial explanatory power according to the recommendations of Hair et al. (2022) (45).

**Table 5:** Structural model quality assessment.

Construct	R^2^	f^2^	VIF	Q^2^
Behavioral Intention (BI)	0.395	GF → BI = 0.018 IV → BI = 0.415 PC → BI = 0.009 TP → BI = 0.000	GF → BI = 1.071 IV → BI = 1.337 PC → BI = 1.759 TP → BI = 1.669	0.281
ChatGPT Usage Behavior (CUB)	0.604	BI → CUB = 1.523	BI → CUB = 1.000	0.536
Guilt feelings (GF)	—	—	—	0.000
Innovativeness (IV)	—	—	—	0.000
Privacy Concern (PC)	—	—	—	0.000
Technophobia (TP)	—	—	—	0.000

Based on Cohen's (1988) guidelines (54), Innovativeness exerted a large effect on Behavioral Intention (f^2^ = 0.415), whereas Guilt Feelings (f^2^ = 0.018), Privacy Concern (f^2^ = 0.009), and Technophobia (f^2^ = 0.000) exhibited negligible effects. Furthermore, Behavioral Intention demonstrated a very large effect on ChatGPT Usage Behavior (f^2^ = 1.523), highlighting its central role in explaining students’ ChatGPT adoption. Assessment of multicollinearity indicated that all variance inflation factor (VIF) values were below the recommended threshold, suggesting that multicollinearity was not a concern in the structural model. Moreover, the Stone–Geisser predictive relevance (Q^2^) values for Behavioral Intention (Q^2^ = 0.281) and ChatGPT Usage Behavior (Q^2^ = 0.536) exceeded zero, indicating that the model possessed satisfactory predictive relevance (45). Finally, common method bias was assessed using Harman's single-factor test. The first factor accounted for 41.26% of the total variance, which is below the recommended threshold of 50% (58), indicating that common method bias is unlikely to have materially affected the study findings.

## Discussion

The present study examined the psychological and individual factors associated with ChatGPT adoption among undergraduate medical students by integrating constructs derived from the Privacy Paradox framework (29), Technostress Theory (30), Affect Theory (31), Diffusion of Innovation Theory (32), and the Theory of Planned Behavior (TPB) (24). Overall, the findings indicated that personal innovativeness was the only antecedent significantly associated with behavioral intention, whereas privacy concern, technophobia, and guilt feelings were not significantly associated with behavioral intention. Furthermore, behavioral intention demonstrated a strong positive association with ChatGPT usage behavior and significantly mediated only the relationship between personal innovativeness and ChatGPT usage behavior. Collectively, these findings suggest that, within the proposed model, innovation-oriented characteristics were more closely associated with students’ intention to use ChatGPT than the psychological barriers examined in the present study.

Beyond testing the proposed hypotheses, the findings contribute to the growing literature on generative AI adoption by demonstrating that psychological barriers and innovation-oriented characteristics were differentially associated with students’ behavioral intention to use ChatGPT. Previous research has primarily explained ChatGPT adoption using traditional technology acceptance models emphasizing cognitive evaluations such as perceived usefulness, perceived ease of use, and performance expectancy (24–28, 59). By integrating complementary theoretical perspectives, the present study provides additional evidence that the relative contribution of psychological and individual factors may vary according to the educational context in which generative AI technologies are adopted.

### Privacy concern and behavioral intention

Contrary to the proposed hypothesis, privacy concern was not significantly associated with behavioral intention to use ChatGPT among undergraduate medical students. Importantly, the observed direction was opposite to the hypothesized negative relationship. Privacy Concern showed a positive bivariate correlation with Behavioral Intention (r = 0.356), and the structural coefficient also remained positive, although substantially smaller and statistically non-significant (*β* = 0.099, *p* = 0.191). Thus, the findings do not simply indicate an absence of association; rather, the hypothesized negative direction was not observed, and the evidence was insufficient to establish a positive association.

One possible explanation is that privacy concern may coexist with continued intention to use ChatGPT. Students who are more attentive to privacy issues may also be more engaged with digital technologies and more aware of ChatGPT, meaning that awareness of potential privacy risks does not necessarily translate into avoidance of the technology. In addition, students may weigh perceived educational benefits against perceived privacy risks when forming their intentions to use ChatGPT. However, because the present study did not directly measure perceived benefits, privacy-risk appraisal, or students’ privacy-management strategies, this interpretation remains tentative.

The difference between the bivariate correlation (r = 0.356) and the smaller structural coefficient (*β* = 0.099) further suggests that part of the observed zero-order association was shared with the other predictors included in the integrated model. Therefore, the positive direction should not be interpreted as evidence that privacy concern positively drives ChatGPT adoption. Instead, it indicates that privacy concern did not demonstrate the hypothesized independent negative association with behavioral intention in the present sample. This finding was not consistent with the expectation derived from the Privacy Paradox framework (29), which proposes that individuals frequently express concerns regarding the collection and use of personal information while simultaneously adopting digital technologies because of their perceived benefits. Within the present study, however, privacy-related concerns were not significantly associated with students’ intention to use ChatGPT. One possible interpretation is that students may evaluate ChatGPT primarily according to its perceived educational value rather than its potential privacy implications. Medical students frequently use digital learning platforms, online educational resources, and cloud-based applications as part of their academic training. Within such learning environments, the perceived benefits of ChatGPT for supporting learning activities may outweigh concerns regarding data privacy when students decide whether to use the technology. Although the present study did not directly assess students’ perceptions of educational benefits or privacy risk, this interpretation is broadly consistent with the Privacy Paradox framework (29), which suggests that adoption decisions often reflect a balance between perceived benefits and perceived risks rather than privacy concerns alone.

Recent research has similarly reported that university students generally acknowledge potential privacy and ethical concerns associated with generative AI while continuing to express favourable attitudes toward its educational use because of its perceived usefulness and learning support (26, 60). Likewise, emerging evidence suggests that students’ acceptance of ChatGPT is more strongly associated with its expected educational value than with concerns related to information privacy or data security (61). Collectively, these observations are consistent with the present findings and suggest that privacy concerns may become less salient when students primarily engage with ChatGPT for educational rather than highly sensitive personal or clinical purposes.

Nevertheless, the absence of a significant association should not be interpreted as evidence that privacy considerations are unimportant in higher education. Rather, the findings indicate that privacy concern was not significantly associated with behavioral intention within the context of the present study. Privacy considerations may assume greater importance in educational or clinical settings involving confidential patient information, electronic health records, or sensitive personal data. Future research conducted across different educational contexts may therefore help clarify the circumstances under which privacy concerns become more strongly associated with students’ adoption of generative AI.

### Technophobia and behavioral intention

Similar to privacy concern, technophobia was not significantly associated with behavioral intention to use ChatGPT among undergraduate medical students. This finding was not consistent with the expectation derived from Technostress Theory (30), which proposes that technology-related anxiety, perceived complexity, and stress may discourage individuals from engaging with new technologies. Within the present study, however, technology-related anxiety did not emerge as a significant correlate of students’ intention to use ChatGPT. One possible interpretation is that the educational environment in which the study was conducted may influence the relevance of technophobia. Undergraduate medical students routinely interact with learning management systems, digital libraries, virtual simulations, electronic resources, and other technology-supported educational platforms throughout their training. Consequently, ChatGPT may be perceived as a continuation of an already established digital learning environment rather than as a completely unfamiliar technology. Although digital literacy and previous experience with AI technologies were not measured directly, this interpretation is consistent with recent evidence indicating that university students increasingly evaluate generative AI according to its educational utility rather than technology-related anxiety (38).

The present findings are also broadly consistent with recent investigations demonstrating that students’ intentions to adopt ChatGPT are more closely associated with cognitive and motivational factors, including performance expectancy, trust, facilitating conditions, and perceived educational value, than with technology-related apprehension. For example, Polyportis and Pahos (2025) reported that performance expectancy and facilitating conditions were more strongly associated with students’ behavioral intention than technology-related concerns (61). Similarly, recent studies examining generative AI adoption have emphasized the importance of perceived usefulness, attitudes, and learning benefits in explaining students’ engagement with ChatGPT (26). Taken together, these findings suggest that the relationship between technophobia and ChatGPT adoption may depend on the educational context and characteristics of the user population. Within digitally mature learning environments, technology-related anxiety may become less prominent when students perceive generative AI as another educational resource rather than as an unfamiliar innovation. Nevertheless, because the present study involved undergraduate medical students from a single institution, additional research across different educational settings and populations is needed to determine whether similar patterns are consistently observed.

### Guilt feelings and behavioral intention

Contrary to the proposed hypothesis, guilt feelings were not significantly associated with behavioral intention to use ChatGPT, although the observed association was negative and approached statistical significance. Thus, while the direction of the relationship was consistent with the expectation derived from Affect Theory (31), the findings did not provide sufficient empirical support for the proposed association within the present sample. According to Affect Theory (31), emotions shape individuals’ evaluations of situations and may subsequently be reflected in their behavioural intentions. Within higher education, guilt feelings associated with generative AI have largely been discussed in relation to concerns about academic integrity, authenticity of learning, and the ethical acceptability of AI-assisted academic work. However, the present findings suggest that such emotional concerns were not consistently associated with students’ intentions to use ChatGPT. Rather than indicating the absence of ethical considerations, the findings may reflect the possibility that students evaluate ethical concerns alongside other educational and practical considerations when deciding whether to engage with generative AI.

One possible interpretation is that students may distinguish between responsible educational use and academically inappropriate use of ChatGPT. For example, using ChatGPT to clarify complex concepts, generate study ideas, summarize learning materials, or receive formative feedback may be perceived differently from using it to complete assignments without appropriate acknowledgement. Recent studies similarly suggest that many university students recognize both the educational value of generative AI and the importance of maintaining academic integrity, resulting in more nuanced attitudes toward AI-assisted learning rather than uniformly negative or positive evaluations (62). The present findings are also broadly consistent with emerging evidence suggesting that students’ adoption of ChatGPT is more closely associated with cognitive evaluations of the technology than with emotional responses alone. Recent investigations have reported that students frequently balance the educational benefits of generative AI against ethical considerations rather than making adoption decisions solely on the basis of emotional concerns (61). This interpretation is also reflected in the present study, where personal innovativeness, rather than guilt feelings, demonstrated a significant positive association with behavioral intention. Nevertheless, the absence of a statistically significant association should not be interpreted as suggesting that ethical concerns are unimportant in higher education. Guilt feelings may become more salient in educational contexts involving high-stakes assessments, formal examinations, or institutional policies that place greater restrictions on AI-assisted learning. As universities continue to develop guidance regarding the responsible use of generative AI, future longitudinal and qualitative research may provide further insight into how students’ emotional responses toward ChatGPT evolve over time.

### Personal innovativeness and behavioral intention

Consistent with the proposed hypothesis, personal innovativeness was positively associated with behavioral intention to use ChatGPT among undergraduate medical students. Among all antecedent variables included in the proposed model, innovativeness demonstrated the strongest association with behavioral intention, suggesting that students who were generally more receptive to exploring new technologies also reported stronger intentions to engage with ChatGPT. This finding is consistent with Diffusion of Innovation Theory (32), which proposes that innovative individuals are typically more willing to experiment with emerging technologies because they perceive novelty and uncertainty as opportunities for learning rather than barriers to adoption.

The present findings are also consistent with previous research identifying personal innovativeness as an important correlate of technology adoption across diverse digital environments (23, 32). Individuals who demonstrate greater openness toward technological innovation are generally more inclined to explore emerging technologies and incorporate them into their routine activities. Within the context of generative AI, this tendency may be particularly relevant because effective use of ChatGPT often requires curiosity, experimentation, and a willingness to adapt to new approaches to learning. Recent empirical evidence similarly highlights the importance of innovation-oriented characteristics in explaining students’ adoption of generative AI. Polyportis and Pahos (2025) reported that students who expressed greater openness toward technological innovation also reported stronger intentions to adopt ChatGPT in higher education (61). Likewise, recent studies suggest that students who perceive generative AI as an opportunity to enhance learning and academic productivity are generally more willing to incorporate these technologies into their educational activities (26, 60). Collectively, these observations are consistent with the present findings and reinforce the relevance of innovation-oriented characteristics in understanding students’ engagement with emerging AI technologies.

An important observation emerging from the present study is that personal innovativeness remained significantly associated with behavioral intention, whereas privacy concern, technophobia, and guilt feelings did not. This pattern suggests that, within the proposed model, students’ openness toward technological innovation was more closely associated with their intention to use ChatGPT than the psychological barriers examined in this study. Rather than indicating that these barriers lack theoretical relevance, the findings suggest that their relative importance may vary according to the educational context and characteristics of the user population.

From a theoretical perspective, these findings support the applicability of Diffusion of Innovation Theory (32) in explaining students’ adoption of generative AI within medical education. While traditional technology acceptance models have primarily emphasized cognitive evaluations of technology (24, 25), the present findings indicate that stable individual characteristics, particularly personal innovativeness, may also contribute to understanding students’ intentions to engage with ChatGPT. Consequently, incorporating innovation-oriented characteristics alongside established technology acceptance constructs may provide a more comprehensive understanding of generative AI adoption in higher education.

### Behavioral intention and ChatGPT usage behavior

Consistent with the proposed hypothesis, behavioral intention was positively associated with ChatGPT usage behavior among undergraduate medical students. This relationship represented the strongest association observed in the structural model (*β* = 0.777) and was accompanied by a very large effect size (*f^2^* = 1.523), indicating that behavioral intention constituted the dominant correlate of ChatGPT usage behavior within the proposed model. Moreover, the model explained 60.4% of the variance in ChatGPT usage behavior (*R^2^* = 0.604), suggesting substantial explanatory power. Collectively, these findings highlight the central position of behavioral intention in understanding students’ reported engagement with ChatGPT.

These findings are consistent with the Theory of Planned Behavior (TPB) (24), which conceptualizes behavioral intention as the construct most closely associated with actual behaviour. According to TPB, behavioural intention reflects an individual's readiness and motivation to perform a particular behaviour and therefore serves as the immediate antecedent of behavioural engagement (24). The present findings support the applicability of TPB in the context of generative AI by indicating that students reporting stronger intentions to use ChatGPT also reported greater engagement with the technology in their academic activities.

The observed association is also consistent with previous technology adoption research demonstrating that behavioural intention remains one of the strongest correlates of actual technology use across a wide range of digital environments (24, 25). Similar findings have been reported in recent studies of generative AI adoption, where behavioural intention consistently demonstrated strong associations with students’ reported use of ChatGPT and related AI technologies (26, 61). Collectively, these observations suggest that behavioural intention continues to occupy a central position in explaining students’ engagement with emerging AI technologies despite the rapid evolution of these systems.

From a theoretical perspective, these findings reinforce the continuing relevance of the Theory of Planned Behavior (24) while also demonstrating the value of integrating TPB with complementary theoretical perspectives. Although generative AI introduces new ethical, psychological, and technological considerations, the present findings suggest that behavioural intention remains closely associated with students’ reported use of ChatGPT. Consequently, incorporating innovation-oriented characteristics alongside established behavioural theories may provide a broader understanding of AI adoption than relying exclusively on traditional technology acceptance constructs.

### Behavioral intention as a mediator

The present study further examined whether behavioral intention mediated the relationships between the proposed antecedent variables and ChatGPT usage behavior. The findings indicated that behavioural intention significantly mediated only the relationship between personal innovativeness and ChatGPT usage behavior, whereas no significant indirect associations were observed for privacy concern, technophobia, or guilt feelings. These findings suggest that, within the proposed model, behavioural intention represented the mechanism through which personal innovativeness was associated with students’ reported use of ChatGPT. This finding is consistent with the Theory of Planned Behavior (24), which proposes that individual beliefs and characteristics are reflected in behaviour primarily through the formation of behavioural intentions. Within the present study, students reporting higher levels of personal innovativeness also reported stronger intentions to use ChatGPT, and these stronger intentions were subsequently associated with greater reported use of the technology. This pattern is theoretically consistent with TPB and further reinforces the central role of behavioural intention within the proposed conceptual framework.

The absence of significant indirect associations for privacy concern, technophobia, and guilt feelings is equally informative. Although these constructs were theoretically expected to contribute to behavioural intention, the findings suggest that they were not associated with ChatGPT usage behavior through this mechanism within the present sample. Rather than questioning the theoretical relevance of these constructs, the findings indicate that their explanatory contribution may differ according to the educational context, institutional environment, and stage of AI adoption. As generative AI becomes increasingly integrated into higher education, students’ adoption decisions may be more closely associated with innovation-oriented characteristics than with concerns regarding privacy, technology-related anxiety, or emotional discomfort.

These findings are also broadly consistent with previous research indicating that behavioural intention frequently represents the principal mechanism linking individual characteristics with technology use (24, 25). Similar mediation patterns have been reported in recent studies of AI adoption, where innovation-oriented characteristics and favourable technology perceptions were associated with actual use primarily through behavioural intention rather than through direct relationships (16, 61). Collectively, these observations reinforce the central role of behavioural intention while also suggesting that not all theoretically relevant antecedents contribute equally to students’ adoption of generative AI.

### Theoretical implications

The present study contributes to the emerging literature on generative AI adoption by extending the theoretical perspectives commonly applied to explain students’ engagement with educational technologies. Previous investigations of ChatGPT adoption have largely relied on traditional technology acceptance models, particularly the Technology Acceptance Model (TAM), the Unified Theory of Acceptance and Use of Technology (UTAUT/UTAUT2), and the Theory of Planned Behavior, with primary emphasis on cognitive evaluations such as perceived usefulness, perceived ease of use, and performance expectancy (24, 25, 27). Comparatively less attention has been devoted to understanding how psychological barriers and stable individual characteristics may jointly relate to students’ adoption of generative AI.

By integrating constructs derived from the Privacy Paradox framework (29), Technostress Theory (30), Affect Theory (31), Diffusion of Innovation Theory (32), and the Theory of Planned Behavior (24), the present study provides a broader theoretical perspective on ChatGPT adoption. Examining these complementary frameworks within a single conceptual model allowed both innovation-oriented characteristics and psychological barriers to be considered simultaneously.

An important theoretical observation is that personal innovativeness, rather than the proposed psychological barriers, demonstrated a significant association with behavioural intention. These findings do not diminish the theoretical importance of privacy, technostress, or affective processes. Instead, they suggest that the relative contribution of these constructs may depend on the educational context in which generative AI is adopted. Within the present study, students’ openness toward technological innovation appeared to be more closely associated with behavioural intention than the psychological barriers examined. This observation highlights the importance of considering contextual variation when applying established technology adoption theories to rapidly evolving AI technologies. The findings also reinforce the continuing relevance of the Theory of Planned Behavior (24). Behavioural intention demonstrated the strongest association with ChatGPT usage behavior and mediated only the relationship between personal innovativeness and technology use. Together, these findings suggest that integrating innovation-oriented characteristics with established behavioural theories may provide a more comprehensive understanding of generative AI adoption than relying exclusively on conventional technology acceptance models.

### Practical implications

The present findings have several practical implications for higher education institutions seeking to integrate generative AI into medical education. First, the significant association between personal innovativeness and behavioral intention suggests that universities may benefit from creating learning environments that encourage students to explore emerging AI technologies with appropriate guidance and critical reflection. Educational initiatives that promote curiosity, experimentation, and digital innovation may support students’ willingness to engage with generative AI, although the present findings do not establish that greater adoption will necessarily result in responsible, appropriate, or effective educational use. Second, the strong association observed between behavioral intention and ChatGPT usage behavior highlights the importance of fostering positive intentions toward the responsible use of AI within educational settings. According to the Theory of Planned Behavior (24), behavioural intention represents the construct most closely associated with behaviour. However, in the present study, behavioral intention and self-reported usage indicate students’ willingness to use ChatGPT and their reported engagement with it; they do not indicate the accuracy, appropriateness, ethicality, or educational effectiveness of that use. Consequently, educational strategies that strengthen students’ confidence in using ChatGPT appropriately while clearly communicating expectations regarding responsible academic practice may facilitate the effective integration of generative AI into teaching and learning.

An important observation emerging from the present study is that privacy concern, technophobia, and guilt feelings were not significantly associated with behavioural intention. Within the present sample and model, these variables therefore did not emerge as statistically significant predictors of students’ intention to use ChatGPT. These findings suggest that institutional efforts may be more effective when directed toward developing students’ AI literacy, critical evaluation skills, and responsible use competencies rather than assuming that privacy concerns, technology-related anxiety, or guilt feelings represent the principal barriers to adoption. Nevertheless, this interpretation should not be understood as diminishing the importance of privacy protection, ethical AI use, or academic integrity. Rather, these issues remain fundamental considerations that should continue to be addressed through institutional policies, curriculum development, and faculty guidance. Given the increasing integration of generative AI into healthcare education, medical schools may also benefit from incorporating AI literacy into competency-based curricula. Such curricula could address not only how to access and use generative AI, but also how to evaluate AI-generated information, recognize its limitations, verify outputs, protect privacy, and comply with academic-integrity requirements. Clear institutional guidance regarding appropriate and inappropriate uses of ChatGPT, together with opportunities for supervised practice and critical reflection, may better prepare future healthcare professionals to engage responsibly with AI technologies in both educational and clinical environments. These recommendations represent implications for educational planning rather than outcomes directly demonstrated by the present study.

### Limitations and future directions

The findings of the present study should be interpreted in light of several limitations. First, the cross-sectional design precludes conclusions regarding temporal ordering or causal relationships among the study variables. Consequently, the directionality of the observed associations cannot be established. Although the proposed conceptual model was theoretically informed, the observed relationships were measured at a single point in time. Future longitudinal research would therefore be valuable for examining how students’ perceptions and engagement with generative AI evolve as these technologies become more widely integrated into higher education. Second, the study was conducted among undergraduate medical students at a single university using a convenience sampling approach. Although King Faisal University attracts students from diverse geographical regions across Saudi Arabia, the findings may not be directly transferable to students from other universities, academic disciplines, healthcare professions, or cultural contexts. Multi-institutional and cross-cultural studies would help determine the consistency of the observed associations across different educational settings. Third, all variables were assessed using self-reported questionnaire measures, which may be influenced by recall bias, social desirability, or subjective perceptions. Although the assessment of common method bias suggested that this was unlikely to represent a substantial concern, future studies may benefit from incorporating objective indicators of AI use, learning analytics, or mixed-method approaches combining quantitative and qualitative data. A further limitation relates to the scope of the proposed conceptual model. The present study focused on four theoretically derived antecedents of ChatGPT adoption. However, previous research has also highlighted the potential relevance of constructs such as AI literacy, trust, facilitating conditions, perceived usefulness, social influence, and institutional AI policies (24, 25, 27, 28, 59). Examining these variables alongside psychological and individual characteristics may contribute to a more comprehensive understanding of generative AI adoption in higher education. Another limitation concerns the possibility of common method variance. Although Harman's single-factor test and full-collinearity VIF assessment did not indicate substantial evidence of severe common method bias, these diagnostics cannot completely rule out method-related variance. All variables were measured through the same self-administered questionnaire, from the same respondents, at a single point in time. Future studies could reduce this concern by incorporating procedural and statistical remedies such as temporal separation of measurements, multiple data sources, objective usage records, or marker-variable approaches where feasible. Finally, the rapid evolution of generative AI technologies should be considered when interpreting the present findings. Students’ perceptions of ChatGPT, institutional policies governing AI use, and educational practices continue to evolve rapidly. Future research should therefore examine whether the relative importance of psychological barriers and innovation-oriented characteristics changes as students gain greater experience with generative AI and as educational institutions continue to refine their approaches to responsible AI integration. Despite these limitations, the present study contributes to the emerging literature by integrating multiple complementary theoretical perspectives to examine psychological barriers, individual characteristics, behavioural intention, and ChatGPT usage behavior within a single conceptual framework. The findings provide a foundation for future investigations examining how technological, psychological, and contextual factors are jointly associated with students’ adoption of generative AI in higher education.

## Conclusion

This study examined psychological and individual factors associated with ChatGPT adoption among undergraduate medical students at King Faisal University, Saudi Arabia. Personal Innovativeness was the only significant predictor of Behavioral Intention, whereas Privacy Concern, Technophobia, and Guilt Feelings were not significant. Behavioral Intention showed a strong positive association with self-reported ChatGPT usage behavior and significantly accounted for the indirect association between Personal Innovativeness and reported usage. These findings highlight the role of individual innovativeness in understanding ChatGPT adoption, while also indicating that psychological barriers may operate differently across contexts. Given the cross-sectional design and single-university convenience sample, future research should use longitudinal, multi-institutional, and objective measures of ChatGPT use, including indicators of responsible and effective educational use.

## Data Availability

The raw data supporting the conclusions of this article will be made available by the authors, without undue reservation.

## Appendix A

**Table A1:** List of constructs and items.

Construct	Items	Description	Source
Privacy Concern	PRC1	My personal information could be misused by ChatGPT system.	Brill et al. (49)
	PRC2	I am concerned that ChatGPT uses my personal information for purposes that I am not being notified of.	
	PRC3	It is dangerous to provide my personal information to ChatGPT.	
Technophobia	TPH1	I believe the evolution of AI is intimidating.	Khasawneh (21)
	TPH2	AI appears threatening to me.	
	TPH3	For reasons unclear to me, AI induces fear.	
Guilt Feeling		This list consists of a number of words and phrases that describe different feelings and emotions you may have had when using ChatGPT. Please reflect on your last week of use of ChatGPT and indicate to what extent you have felt this way when using the website: (1 = Very slightly or not at all 7 = Extremely)	Masood et al. (22)
	GFE1	Guilty	
	GFE2	Ashamed	
	GFE3	Blameworthy	
Innovativeness	INO1	I like to experiment with new technologies.	Agarwal and Prasad (23)
	INO2	Among my peers, I am usually the first to try out new information technologies.	
	INO3	In general, I would not hesitate to try out new information technologies.	
Behavioral intention UTAUT2	BIT1	I intend to continue using ChatGPT in the future.	Venkatesh et al. (25)
	BIT2	I will always try to use ChatGPT in my daily life.	
	BIT3	I plan to continue to use ChatGPT frequently.	
Behavior	BEH1	How often do you use ChatGPT?	McLean and Osei-Frimpong (50) Aparicio et al. (51)
	BEH2	I consider myself a frequent user of ChatGPT.	
